# Culture-Competent SARS-CoV-2 in Nasopharynx of Symptomatic Neonates, Children, and Adolescents

**DOI:** 10.3201/eid2610.202403

**Published:** 2020-10

**Authors:** Arnaud G. L’Huillier, Giulia Torriani, Fiona Pigny, Laurent Kaiser, Isabella Eckerle

**Affiliations:** Geneva University Hospitals and Faculty of Medicine, University of Geneva, Geneva, Switzerland

**Keywords:** coronavirus disease, 2019 novel coronavirus disease, COVID-19, SARS-CoV-2, severe acute respiratory syndrome coronavirus 2, respiratory diseases, zoonoses, viruses, neonates, children, adolescents, viral shedding, Switzerland

## Abstract

Children do not seem to drive transmission of severe acute respiratory syndrome coronavirus 2 (SARS-CoV-2). We isolated culture-competent virus in vitro from 12 (52%) of 23 SARS-CoV-2–infected children; the youngest was 7 days old. Our findings show that symptomatic neonates, children, and teenagers shed infectious SARS-CoV-2, suggesting that transmission from them is plausible.

Children are underrepresented in coronavirus disease (COVID-19) case numbers (*1*,*2*). Severity in most children is limited, and children do not seem to be major drivers of transmission (*3*,*4*). However, severe acute respiratory syndrome coronavirus 2 (SARS-CoV-2) infects children of all ages (*1*,*3*). Despite the high proportion of mild or asymptomatic infections (*5*), they should be considered as transmitters unless proven otherwise. To address this point, the laboratory of the Geneva University Hospitals and Faculty of Medicine, University of Geneva (Geneva, Switzerland), used cell culture to systematically assess cultivable SARS-CoV-2 in the upper respiratory tract (URT) of 23 children with COVID-19.

All nasopharyngeal specimens (NPS) were collected with a flocked swab in universal transport medium (Floqswab; Copan, https://www.copangroup.com) and tested for SARS-CoV-2 by reverse transcription PCR during January 25–March 31, 2020 (Appendix). We seeded Vero E6 cells at 8 × 10^4^ cells/well in a 24-well plate and inoculated them with 200 μL of viral transport medium the following day. Cells were inoculated for 1 h at 37°C; inoculum was removed; cells were washed once with phosphate buffered saline; and regular cell growth medium containing 10% fetal calf serum was added. We observed cells on days 2, 4, and 6 for cytopathic effect (CPE) by light microscopy. We harvested supernatant at first observation of CPE or, if no CPE occurred, on day 6. For a second passage, we transferred 20 μL supernatant of CPE-positive specimens onto new Vero E6 cells. We collected supernatant after inoculation and on observation of CPE and confirmed isolation of replication competent SARS-CoV-2 by an increase in viral RNA (Appendix).

Of 638 patients <16 years of age, 23 (3.6%) tested positive for SARS-CoV-2. Median age was 12.0 years (interquartile range [IQR] 3.8–14.5 years, range 7 days–15.9 years). Thirteen patients had an URT infection; 2 each had fever without source and pneumonia (Table). Samples were collected a median of 2 (IQR 1–3) days after symptom onset. Median viral RNA load at diagnosis was 3.0 × 10^6^ copies/mL (mean 4.4 × 10^8^ [IQR 6.9 × 10^3^–4.4 × 10^8^] copies/mL; peak 5.3 × 10^9^ copies/mL).

**Table:** Characteristics and results of children <16 years of age with coronavirus disease, Geneva University Hospitals and Faculty of Medicine, University of Geneva, Switzerland, January 25–March 31, 2020*

Patient	Age	Days from symptom onset to diagnosis	Clinical diagnosis	Hospital admission	Viral RNA copies/mL	Isolate
1	12.6 y	1	URTI	No	2.8 × 10^7^	Negative
2	5.7 y	1	URTI	No	1.8 × 10^6^	Negative
3	14.8 y	1	URTI	No	9.9 × 10^6^	Positive
4	12.0 y	2	Obstructive bronchitis	No	6.9 × 10^3^	Negative
5	3.9 y	4	URTI	No	4.5 × 10^3^	Negative
6	13.9 y	2	Pneumonia	Yes	8.6 × 10^7^	Positive
7	9.0 y	2	Croup	No	6.2 × 10^3^	Negative
8	10.1 y	3	URTI	No	3.3 × 10^5^	Negative
9	3 mo	Not reported	Not reported	Yes	2.8 × 10^2^	Negative
10	2.2 y	Not reported	Not reported	Yes	5.9 × 10^2^	Negative
11	8.4 y	1	URTI	No	5.6 × 10^8^	Negative
12	7 d	1	URTI	No	1.3 × 10^8^	Positive
13	12.9 y	4	Pneumonia	Yes	4.2 × 10^3^	Negative
14	15.7 y	Not reported	Not reported	No	2.5 × 10^4^	Negative
15	12.3 y	2	Influenza-like illness	No	1.1 × 10^9^	Positive
16	15.9 y	1	Fever without source	Yes	2.2 × 10^8^	Positive
17	1 mo	0	Fever without source	Yes	5.3 × 10^9^	Positive
18	2 mo	1	URTI	No	4.4 × 10^8^	Positive
19	5.9 y	1	URTI	No	1.6 × 10^9^	Positive
20	15.9 y	2	URTI	No	6.8 × 10^8^	Positive
21	14.4 y	5	URTI	Yes	1.4 × 10^5^	Positive
22	14.6 y	3	URTI	No	1.2 × 10^4^	Positive
23	14.4 y	2	URTI	No	3.0 × 10^6^	Positive

*URTI, upper respiratory tract infection.

We isolated SARS-CoV-2 from 12 (52%) children. We determined SARS-CoV-2 isolation by presence of CPE and increased viral RNA in the supernatant (Table; Appendix Figure). SARS-CoV-2 replication in all 12 positive isolates was confirmed by a second passage.

We isolated virus from children of all ages; the youngest was 7 days of age. Median viral load was higher for patients with isolation (1.7 × 10^8^ [mean 7.9 × 10^8^, IQR 4.7 × 10^6^–1.0 × 10^9^] copies/mL) than for those without isolation (6.9 × 10^3^ [mean 5.4 × 10^7^, IQR 4.2 × 10^3^–1.8 × 10^6^] copies/mL; p = 0.002) (Figure). Sex, age, duration of symptoms, clinical diagnosis, symptoms, and likelihood of admission did not differ between patients with and without isolation (Appendix Table).

**Figure:**
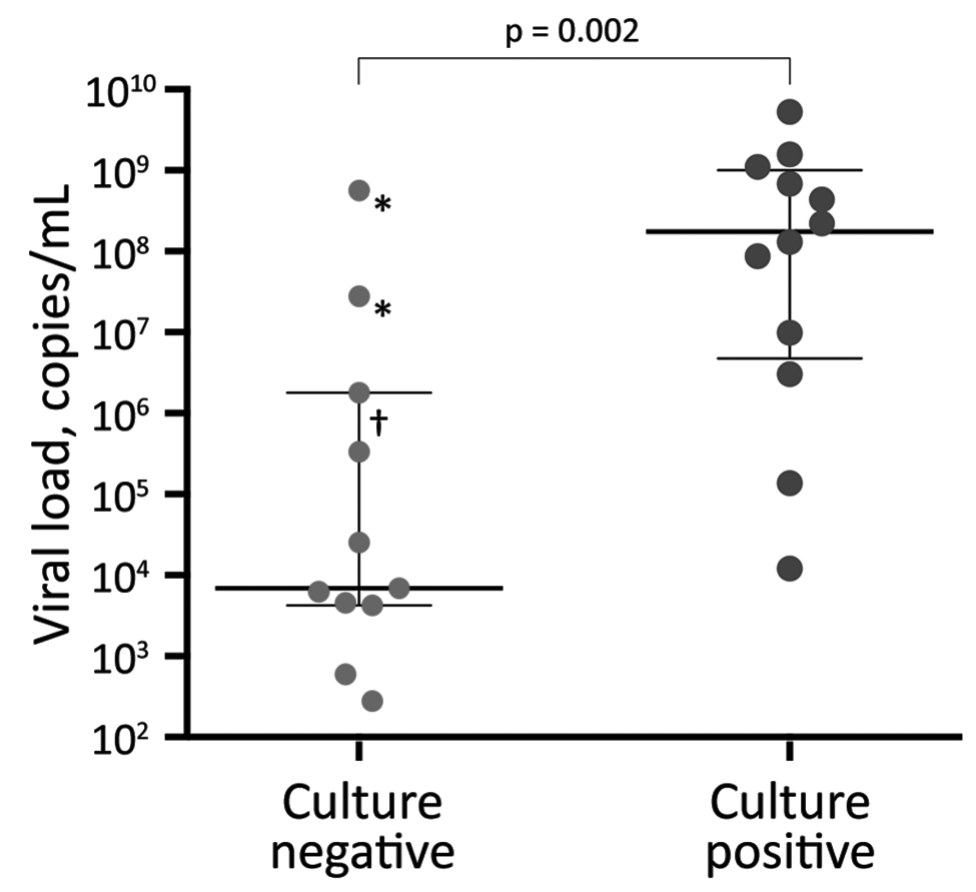
Severe acute respiratory syndrome coronavirus 2 initial RNA copy numbers from nasopharyngeal swabs of culture-negative and culture-positive specimens from children <16 years of age, Geneva University Hospitals, Geneva, Switzerland, January 25–March 31, 2020. Thick horizontal bars indicate median RNA; thin horizontal bars indicate interquartile range. Asterisk (*) indicates specimen collected outside the institution, suggesting a longer time to freezing at –80°C; dagger (†) indicates specimen with ≈48 hours from specimen collection to freezing at –80°C.

Our data show that viral load at diagnosis is comparable to that of adults (*6*,*7*) and that symptomatic children of all ages shed infectious virus in early acute illness, a prerequisite for further transmission. Isolation of infectious virus was largely comparable with that of adults, although 2 specimens yielded an isolate at lower viral load (1.2 × 10^4^ and 1.4 × 10^5^ copies/mL) (*6*).

A limitation of our study was the small number of children assessed. However, although the Canton of Geneva was a region severely affected by SARS-CoV-2 (*8*), only 23 cases were diagnosed in children at our hospital during the study period. These findings confirm that children are not a major risk group for COVID-19. Another limitation is our reliance solely on leftover material initially received for routine diagnostic purposes that we retrospectively analyzed. Using such specimens has several disadvantages: preanalytic quality of specimens could be affected by suboptimal times between sample collection and storage at −80°C because of transport and diagnostic processing time, resulting in loss in infectivity and failure of virus isolation even in the presence of high viral load. Therefore, our findings probably underestimate the true rate of infectious virus presence in symptomatic children, and we cannot comment whether our data reflect the rates of infectious virus shedding in the community. Because of the limited leftover volume of the specimens, we were unable to further investigate the quantity of infectious viral particles. Most patients were managed as outpatients and self-isolated at home, so no consecutive sampling was possible to assess infectious virus in multiple samples over the course of disease.

SARS-CoV-2 viral load and shedding patterns of culture-competent virus in 12 symptomatic children resemble those in adults. Therefore, transmission of SARS-CoV-2 from children is plausible. Considering the relatively low frequency of infected children, even in severely affected areas, biological or other unknown factors could lead to the lower transmission in this population. Large serologic investigations and systematic surveillance for acute respiratory diseases and asymptomatic presentations are needed to assess the role of children in this pandemic.

Appendix Additional methods for a study of SARS-CoV-2 in symptomatic neonates, children, and adolescents.
