# Supplementary material for: Culture-Competent SARS-CoV-2 in Nasopharynx of Symptomatic Neonates, Children, and Adolescents
Source: Emerg Infect Dis. 2020 Oct;26(10):2494–7. doi: 10.3201/eid2610.202403 (PMC7510703; doi:10.3201/eid2610.202403)
Supplement: Appendix — Additional methods for a study of SARS-CoV-2 in symptomatic neonates, children, and adolescents. [file 20-2403-Techapp-s1.pdf]

Article DOI: https://doi.org/10.3201/eid2610.202403

Culture-Competent SARS-CoV-2 in Nasopharynx of Symptomatic Neonates, Children, and Adolescents

Appendix

Supplementary Methods

Our region had a SARS-CoV-2 outbreak, with an estimated 800 cases/100,000 inhabitants and reaching a seroprevalence of around 10% in the general population in early May (*1*). Among 638 patients <16 years old tested for SARS-CoV-2 by RT-PCR on nasopharyngeal swabs (NPS) between January 25 and March 31, 2020, 23 (3.6%) tested positive. Patients were either seen at the pediatric emergency room of the Geneva University Hospitals, or samples were received by the laboratory from other healthcare facilities as part of its function as the Swiss national reference laboratory for Emerging Viral Diseases at the Geneva University Hospitals.

All NPS specimens were collected with a flocked swab in universal transport medium (Floqswab, Copan, Italy) and tested for SARS-CoV-2 according to manufacturers' instructions on various platforms over the course of the outbreak, including initially in house methods using eMAG extraction (bioMérieux, France) and Charité RT-PCR protocol (*2*), then BD SARS-CoV-2 reagent kit for BD Max system (Becton, Dickinson and Co, U.S.) and Cobas 6800 SARS CoV2 RT-PCR (Roche, Switzerland). Several diagnostic systems were used in parallel during the course of the pandemic to fulfill the steeply increasing diagnostic demand. All samples, both extracted RNA as well as remaining original specimens, were stored at −20°C and −80°C, respectively. For this study, RNA extracts of all samples were re-run with the E-gene assay (TibMolBiol, Berlin, Germany) on a Roche Light Cycler 480 (Roche Switzerland) according to manufacturers' instructions, by using in vitro transcribed RNA for quantification (European Virus Archive) (*3*). Cell culture supernatant was isolated by manual extraction with Machery & Nagel Kit (Düren, Germany) and quantified by the same assay.

Clinical data of study patients were retrieved after approval by the institutional review board (Commission Cantonale d'Ethique de la Recherche [CCER] protocol 2020–00835) and documented parental consent in the medical charts.

Statistics were performed using SPSS version 23.0 (IBM Corp., 21 Armonk, NY, USA). Figures were made using GraphPad version 7.0 (LaJolla, CA, 22 USA).

References

1. Stringhini S, Wisniak A, Piumatti G, Azman AS, Lauer SA, Baysson H, et al. Seroprevalence of anti-SARS-CoV-2 IgG antibodies in Geneva, Switzerland (SEROCoV-POP): a population-based study. Lancet. 2020;S0140-6736(20)31304-0 [Epub ahead of print]. PubMed https://doi.org/10.1016/S0140-6736(20)31304-0

2. Corman VM, Landt O, Kaiser M, Molenkamp R, Meijer A, Chu DK, et al. Detection of 2019 novel coronavirus (2019-nCoV) by real-time RT-PCR. Euro Surveill. 2020;25. PubMed https://doi.org/10.2807/1560-7917.ES.2020.25.3.2000045

3. European Virus Archive Global (EVAg) [cited 2020 Apr 24]. https://www.european-virus-archive.com

Appendix Table. Comparison of patients with and without successful virus isolation*

Variable	Culture negative, n = 11	Culture positive, n = 12	p value
Age, y, median (IQR)	9.0 (3.9–12.6)	14.1 (1.6–15.6)	0.242
Female sex, no. (%)	5 (45)	7 (58)	0.537
Duration of symptoms until diagnosis, d, median (IQR)	2.0 (1.0–3.8)	1.5 (1.0–2.0)	0.373
Admission, no. (%)	3 (27)	3 (25)	0.999
Diagnosis, no. (%)			0.193
Upper respiratory tract infection	5 (45)	8 (73)	
Pneumonia	1 (9)	1 (8)	
Fever without source	1 (9)	1 (9)	
Influenza-like illness	0	0	
Obstructive bronchitis	1 (9)	0	
Croup	1 (9)	0	
Not reported	3 (27)	0	
Reported symptoms†			
Cough	7 (88)	9 (75)	0.619
Nasal discharge	7 (88)	9 (75)	0.619
Shortness of breath	4 (50)	3 (25)	0.356
Dysphagia	3 (38)	3 (25)	0.303
Fever	6 (75)	9 (75)	0.999
Arthralgia	4 (50)	1 (8)	0.062
Myalgia	5 (63)	2 (17)	0.071
Nausea	0	3 (25)	0.057
Vomiting	0	2 (17)	0.495
Diarrhea	0	5 (42)	0.055
Abdominal pain	2 (25)	4 (33)	0.214
Anosmia	1 (13)	3 (25)	0.535
Headache	4 (50)	6 (50)	0.240
Fatigue	4 (50)	8 (67)	0.648
Rash	1 (13)	3 (25)	0.619

*IQR: interquartile range
†For 3 symptomatic patients in the culture negative group, diagnosis and exact symptoms were not reported.

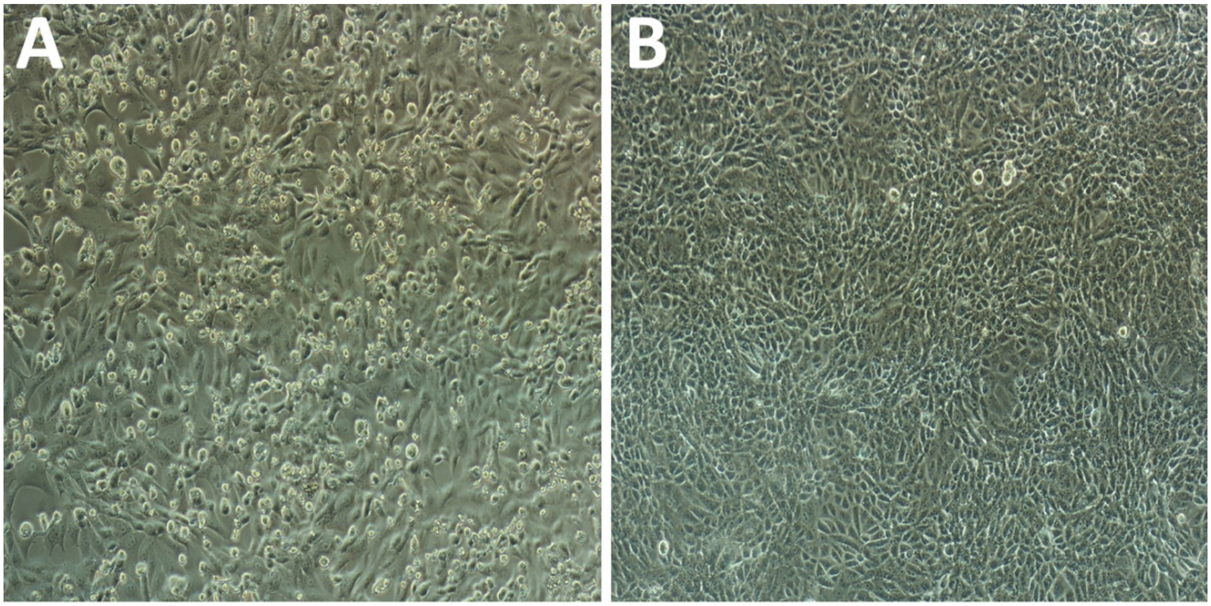

Appendix Figure. Cytopathic effect on VeroE6 cells inoculated at the 2nd passage with severe acute respiratory syndrome coronavirus 2 isolated from a nasopharyngeal swab sample from a child with coronavirus disease 2 days after infection (A) and uninfected control (B).
